# Supplementary material for: PauseNørd Pilot Study: Exploring the Implementation of Mini Movement Breaks in University Lectures
Source: Int J Environ Res Public Health. 2025 May 7;22(5):739. doi: 10.3390/ijerph22050739 (PMC12111276; doi:10.3390/ijerph22050739)
Supplement: Supplementary file 1 [file ijerph-22-00739-s001.zip › ijerph-3481785-supplementary.pdf]

International Journal of
Environmental Research
and Public Health

MDPI

Supplementary materials: Questionnaires and Interviews

A copy of a SurveyXact questionnaire Q1 can be found at this link:
https://www.survey-xact.dk/LinkCollector?key=HVA41H9LLN35

The paper copy of the questionnaire Q1 is as follows:

Participant questionnaire

PauseNørd

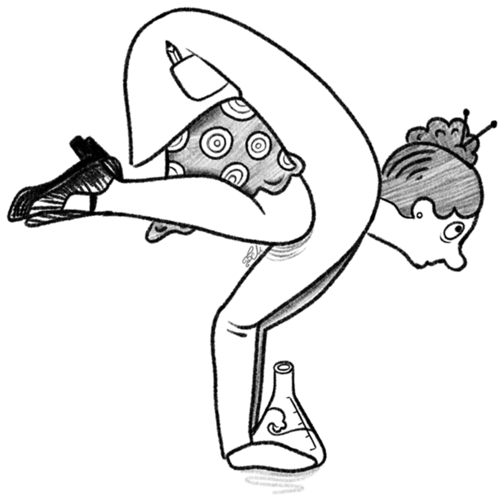

Artist: Sissel Helbæk Mogensen

Q1 – pre and post

Please indicate from 0 (lowest) to 10 (highest) your overall:

Level of mental alertness (how you are able to think fast and notice things)

0	1	2	3	4	5	6	7	8	9	10

Level of concentration (how are your able to focus your attention without getting distracted / mind wandering)

0	1	2	3	4	5	6	7	8	9	10

Level of enjoyment related to the class

0	1	2	3	4	5	6	7	8	9	10

Level of motivation for following the class

0	1	2	3	4	5	6	7	8	9	10

Level of sleepiness (how you feel tired and with a desire to sleep)

0	1	2	3	4	5	6	7	8	9	10

Level of cognitive fatigue (how you feel mentally exhausted because of prolonged demanding tasks)

0	1	2	3	4	5	6	7	8	9	10

Level of restlessness due to prolonged sitting (how you feel a mental and/or physical discomfort caused by sitting for a long period)

0	1	2	3	4	5	6	7	8	9	10

Level of positive social interactions within the class

0	1	2	3	4	5	6	7	8	9	10

Positive mood

0	1	2	3	4	5	6	7	8	9	10

A copy of a SurveyXact questionnaire Q2 (methodology M3) is copied below:

Participant questionnaire

PauseNørd Study

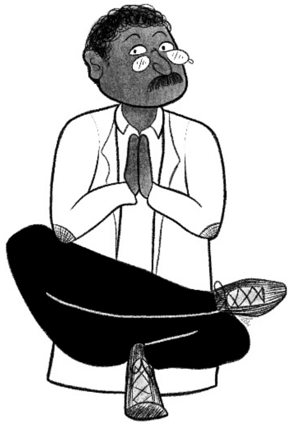

Artist: Sissel Helbæk Mogensen

Q2 - post

Please indicate from 0 (lowest) to 10 (highest) how was <u>throughout the class</u> your overall:

Level of mental alertness (how you are able to think fast and notice things)

0	1	2	3	4	5	6	7	8	9	10

Level of concentration (how are your able to focus your attention without getting distracted / mind wandering)

0	1	2	3	4	5	6	7	8	9	10

Level of enjoyment related to the class

0	1	2	3	4	5	6	7	8	9	10

Level of motivation for following the class

0	1	2	3	4	5	6	7	8	9	10

Level of sleepiness (how you feel tired and with a desire to sleep)

0	1	2	3	4	5	6	7	8	9	10

Level of cognitive fatigue (how you feel mentally exhausted because of prolonged demanding tasks)

0	1	2	3	4	5	6	7	8	9	10

Level of restlessness due to prolonged sitting (how you feel a mental and/or physical discomfort caused by sitting for a long period)

0	1	2	3	4	5	6	7	8	9	10

Level of positive social interactions within the class

0	1	2	3	4	5	6	7	8	9	10

Positive mood

0	1	2	3	4	5	6	7	8	9	10

A copy of the interview guide for students:

Interview - students	Questions
**Preparation**	▪ **Audio-recorder**<br>▪ **Information on the processing of personal data – PauseNørd Pilot Study**
**Introductory part**	▪ Welcoming<br>▪ Short description of the topic<br>▪ Introduction of the rules:<br>o Information on the processing of personal data – PauseNørd Pilot Study<br>o audio recording<br>o acknowledging diverse opinions in case more than one person present
**Transitory question**	▪ Do you take breaks when studying by yourself? And do you structure your breaks?
**Main questions**	**Acceptability** What did you think when PN was first introduced to you? What are the positive and negative experiences related to PN? What made the experience with PN easier/more positive? What made the experience with PN more difficult/ negative?<br><br>**Efficacy:** Did PN have any effect on you?<br><br>**Practicality:** What are your thoughts about incorporating more PN breaks in your lecture?<br><br>**Expansion:** Do you think that the PN breaks could be implemented in all kinds of lectures? Are there factors that may inhibit or facilitate its implementation on a larger scale?
**Closure**	▪ Do you have anything else in mind you would like to share before we end the interview?<br>▪ Closing of the interview, thanks, and farewells.

A copy of the interview guide for lecturers:

<table>
<tr><th>Interview - Lecturers</th><th>Questions</th></tr>
<tr><td>Preparation</td><td>Audio-recorder
Information on the processing of personal data – PauseNørd Pilot Study</td></tr>
<tr><td>Introductory part</td><td>Welcoming
Short description of the topic
Introduction of the rules:
Information on the processing of personal data – PauseNørd Pilot Study
audio recording
acknowledging diverse opinions in case more than one person present
Round of introductions
Names, their experience in teaching, kind and number of courses, number of students</td></tr>
<tr><td>Transitory question</td><td>Have you noticed drops in attention among the students during the lectures? Is there any strategy you use?</td></tr>
<tr><td>Main questions</td><td>Acceptability: What was your experience of PN as a lecturer? And what did you observe from the students, what were the students’ attitudes and reactions? (both positive and negative experiences).

Efficacy: Did PN have any effect on you and your teaching?

Practicality: How feasible is the delivery of PN in your lectures? (Consider factors such as lecture duration, frequency of breaks, and ease of implementation within the existing lecture structure). Do you think it makes sense to incorporate the PN breaks in your lectures? How easily can these breaks be accommodated within the lecture schedule?

Expansion: Do you think that the PN breaks could be implemented in all kinds of lectures? Are there factors that may inhibit or facilitate its implementation on a larger scale?</td></tr>
</table>

**Closure**	▪ Would you have any other specific recommendation or issue that wasn't discussed yet?<br>▪ Closing of the interview, thanks, and farewells.
